# Pathobiont and symbiont contribute to microbiota homeostasis through Malpighian tubules–gut countercurrent flow in *Bactrocera dorsalis*

**DOI:** 10.1093/ismejo/wrae221

**Published:** 2024-11-12

**Authors:** Yanning Liu, Rengang Luo, Shuai Bai, Bruno Lemaitre, Hongyu Zhang, Xiaoxue Li

**Affiliations:** National Key Laboratory for Germplasm Innovation and Utilization for Fruit and Vegetable Horticultural Crops, Hubei Hongshan Laboratory, China–Australia Joint Research Centre for Horticultural and Urban Pests, Institute of Urban and Horticultural Entomology, College of Plant Science and Technology, Huazhong Agricultural University, Wuhan, Hubei Province 430070, China; National Key Laboratory for Germplasm Innovation and Utilization for Fruit and Vegetable Horticultural Crops, Hubei Hongshan Laboratory, China–Australia Joint Research Centre for Horticultural and Urban Pests, Institute of Urban and Horticultural Entomology, College of Plant Science and Technology, Huazhong Agricultural University, Wuhan, Hubei Province 430070, China; National Key Laboratory for Germplasm Innovation and Utilization for Fruit and Vegetable Horticultural Crops, Hubei Hongshan Laboratory, China–Australia Joint Research Centre for Horticultural and Urban Pests, Institute of Urban and Horticultural Entomology, College of Plant Science and Technology, Huazhong Agricultural University, Wuhan, Hubei Province 430070, China; Global Health Institute, School of Life Science, École Polytechnique Fédérale de Lausanne (EPFL), Station 19, 1015, Lausanne, Switzerland; National Key Laboratory for Germplasm Innovation and Utilization for Fruit and Vegetable Horticultural Crops, Hubei Hongshan Laboratory, China–Australia Joint Research Centre for Horticultural and Urban Pests, Institute of Urban and Horticultural Entomology, College of Plant Science and Technology, Huazhong Agricultural University, Wuhan, Hubei Province 430070, China; National Key Laboratory for Germplasm Innovation and Utilization for Fruit and Vegetable Horticultural Crops, Hubei Hongshan Laboratory, China–Australia Joint Research Centre for Horticultural and Urban Pests, Institute of Urban and Horticultural Entomology, College of Plant Science and Technology, Huazhong Agricultural University, Wuhan, Hubei Province 430070, China

**Keywords:** microbiota, pathobiont, symbiont, Malpighian tubules, countercurrent flow, duox, peristalsis, Bactrocera dorsalis

## Abstract

Host–gut microbiota interactions are more complex than good or bad. Both gut symbiotic bacteria and pathobionts can provide essential functions to their host in one scenario and yet be detrimental to host health in another. So, these gut-dwelling bacteria must be tightly controlled to avoid harmful effects on the host. However, how pathobionts and other symbiotic bacteria coordinate to establish a host immune defense system remains unclear. Here, using a Tephritidae fruit fly *Bactrocera dorsalis*, we report that both pathobionts and other gut symbiotic bacteria release tyramine, which is recognized by the host insects. These tyramines induce the formation of insect-conserved Malpighian tubules–gut countercurrent flow upon bacterial infection, which requires tyramine receptors and aquaporins. At the same time, pathobionts but not gut symbiotic bacteria induce the generation of reactive oxygen species, which are preserved by the countercurrent flow, promoting bacteria elimination through increasing gut peristalsis. More importantly, our results show that the Malpighian tubules–gut countercurrent flow maintains proper microbiota composition. Our work suggests a model where pathobiont-induced reactive oxygen species are preserved by Malpighian tubules–gut countercurrent flow involving both pathobionts and symbiotic bacteria. Furthermore, our work provides a Malpighian tubules–gut interaction that ensures efficient maintenance of the gut microbiota.

## Graphical Abstract

**Graphical Abstract:**
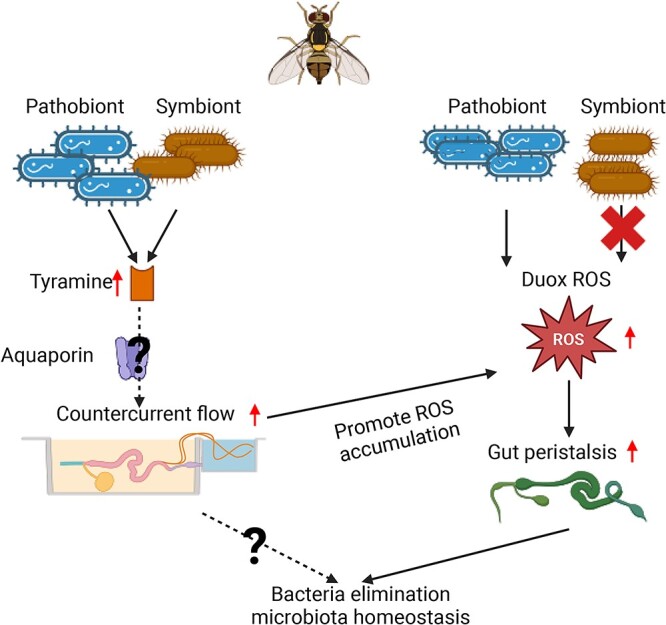

## Introduction

Living in a microbe-rich environment, insects, such as Dipteran species, interact with a broad range of symbionts, opportunists, and pathogens. This diversity of cohabitants has likely shaped the sophisticated insect immune system, which must be capable to combat pathogens while promoting the growth of beneficial microbes. Studies mostly done in *Drosophila melanogaster* have shown the multiple roles of the gut microbiota on insect health by promoting host development under poor nutrient conditions, providing vitamins, or preventing infection [1–3]. Over-growth of one or more normal species making up the microflora causes intestinal dysfunction and diseases, affecting gut homeostasis and shortens lifespan [1, 4]. Accumulating evidence has shown that microbiota–host interactions are complex that cannot be simplified by good or bad.

Whereas many gut-dwelling bacteria generally benefit the host’s physiology, they can also be detrimental and lead to host pathogenesis in some cases. These bacteria are called pathobiont or pathogenic potential [5]. In *D. melanogaster*, the over-growth of a gut-dwelling bacteria, *Gluconobacter morbifer* G707T, induces intestinal dysbiosis, which causes gut cell apoptosis [6, 7]. These studies suggested gut microbiota must be tightly controlled by the host immune system, which is complex, encompassing various constitutive and inducible defenses. First, physical barriers protect the gut epithelium from direct contact with bacteria. These include the cuticle that lines the foregut and hindgut, and a chitinous layer called the peritrophic membrane (PM) that lines the midgut [8, 9]. The PM forms a permeable porous matrix to allow the digestive process while separating bacteria and harmful particles from the gut epithelium [10]. An acidic region in the middle part of the midgut also contributes to eliminating ingested bacteria [11, 12]. Second, the mechanical flushing of pathogens out of the intestine by peristalsis is thought to be a vital host defense mechanism. Peristalsis involves a wave-like longitudinal and circular muscle contraction that moves food and bacteria through the digestive tract [13]. The waves can be short, regional reflexes or long, continuous contractions that travel the whole gut. In insects, as in mammals, gastrointestinal motility is controlled by the secretory products of enteroendocrine cells as well as enteric and central nervous systems [14, 15]. Ingestion of bacteria promotes strong midgut visceral contractions, a process involving reactive oxygen species (ROS) production by the NADPH Duox by enterocytes [16, 17].

Ingestion of bacteria triggers the production of hypochlorous acid (HOCl) by Duox. Its microbicidal role is thought to be crucial for insects to survive oral bacteria infection [18, 19]. Production of ROS by Duox is achieved through recognizing pathogen and pathobiont-released uracil, but not the symbiotic bacteria via the Gαq-PLCβ-Ca_2_^+^ pathway and activation of MEKK1-MKK3-p38 MAPK pathways [6, 18, 20]. In addition, lipid catabolism also upregulated transcription of the *Duox* gene^1^. Although initial studies have shown that Duox-derived HOCl has a direct bactericidal effect, other studies revealed another role for Duox in gut homeostasis. As described above, Duox is also involved in peristalsis but also contributes to the increased stem cell proliferation observed upon bacterial infection [16, 17, 21, 22].

We have recently shown that Malpighian tubules are required for gut homeostasis post oral infection [23]*.* Malpighian tubules form the insect kidney, regulating osmolarity/water homeostasis [10]. Malpighian tubules are connected at the posterior end of the midgut, where they generate primary urine [24]. Studies done in several insect species, particularly locusts, have revealed the existence of counterflows that can flush Malpighian tubule liquid toward the anterior parts of the gut [25, 26]. The existence of retrograde fluid flow in the insect gut is possibly due to the peritrophic matrix that transversally compartmentalizes the midgut. According to this model, food and bacteria transit forward along the gut in the endoperitrophic space formed by the PM, whereas the countercurrent flow initiated by Malpighian tubules takes place in the ectoperitrophic space between the PM and the epithelium. Our previous study reveals that this countercurrent flow increases upon bacterial oral infection and brings the tubules-produced Jak–STAT pathway ligand Unpaired 3 (Upd3) to the anterior part of the midgut to promote an increased epithelium renewal. The observation that this counterflow is increased upon bacterial infection suggested that it could play an important role in host defense.

Here, we used the Tephritidae fruit fly *Bactrocera dorsalis*, an invasive dipteran *Bactrocera* pest that has continued to pose a significant threat to the fruit and vegetable industry, causing enormous economic losses worldwide [27]. *B. dorsalis* larva lives in rotten fruits and owns a complex gut microbiota, making it a good model for gut microbiota study [28]. Here, we show that pathobiont and other symbiotic bacteria-derived tyramine induce the formation of Malpighian tubules–gut countercurrent flow, which requires aquaporin Prip. This flow ensures the accumulation of ROS produced by Duox upon recognition of pathobiont. Our results show that this countercurrent flow is required for increased gut peristalsis and gut microbiota homeostasis. Collectively, our data highlights the roles of pathobiont and other symbiotic bacteria in the early stage gut immune response. Our study also reveals the importance of Malpighian tubules–gut countercurrent flow in gut microbiota maintenance.

## Materials and methods

### Insect rearing

The *B. dorsalis* were raised at the Institute of Horticultural and Urban Entomology, Huazhong Agricultural University (Wuhan, China), under conditions of 28 ± 1°C and 70%–80% relative humidity, with a 14 h light/10 h dark cycle. Larvae were raised on an artificial diet constituting of 300 g banana, 300 g corn meal, 60 g yeast, 60 g sucrose, 1.2 g sodium paraben methyl ester, 60 g tissue paper, 2.4 ml hydrochloric acid, 600 ml water. Upon emergence, the adult flies were transferred to cages measuring 30 × 30 × 30 cm and nourished with a synthetic food source. The artificial diet made of 10% sucrose, 3.4% yeast, 1% agar, 1.7% honey, and supplemented with 1.6 g/L sodium paraben methyl ester. Female flies that emergence for 5 to 8 days were used for all experiments.

### Oral infection and bacteria persistence

*Providencia rettgeri*, *Citrobacter koseri*, and *Enterobacter hormaechei* were cultured in Luria–Bertani (LB) medium for 14 h at 37°C overnight, 220 rpm from single colony. Bacteria culture were harvested by centrifuge (4200 rpm, 4°C, 10 min). By combining an equal volume of an overnight-grown culture of *P. rettgeri* and commensal bacteria (having an optical density of OD600 = 200) with a 5% sucrose solution in a 1:1 ratio, the infection solution was prepared, which was then applied to cover the surface of the artificial diet. Adult female *B. dorsalis*, aged between five and eight days, were subjected to a 2 h starvation period in a plastic box maintained at 28°C, then supplied with the infection solution. For all experiments in this paper, the flies were subjected to a 2 h infection and then switched to a clean fly food for the following sampling. The time points mentioned in the paper indicate the time post the 2 h infection process. For the bacteria persistence experiment, the flies were firstly washed twice in 75% ethanol for 30 s, then washed using phosphate-buffered saline (PBS) for 30 s. The flies were then homogenized in a 2 ml freezing tube containing 200 μl of LB solution and 3 steel beads. For feces samples collection, flies were moved to a 50 mL falcon tube after infection and then feces were washed and collected using LB at desired time point. The samples were serially diluted and dilutions were then plated (2 μl) onto the LB plates. The plates were cultivated overnight at 37°C (*P. rettgeri*). In the vitamin C feeding experiment (Coolaber, China), the infection solution was supplemented with 50 mg/ml of vitamin C. Similarly, for the Tyramine feeding experiment (MACKLIN, China), the infection solution was enriched with 70 mM of Tyramine.

### Countercurrent flow measurement

Two small wells were dug next to each other in a polydimethylsiloxane (PDMS) plate. A mixture containing an equal proportion of Schneider’s insect medium (Pricella, China) and insect saline buffer was dispensed into the two wells. Brilliant Blue solution (Sigma, Germany) was added into one of the wells to make the final concentration of 0.5 g/L. The gut and Malpighian tubules of adult female *B. dorsalis* were dissected. The Malpighian tubules were placed in the well containing Brilliant Blue dye, while the gut was put into the other well. The PDMS plate was then sealed with mineral oil (Beyotime, China) to prevent tissue evaporation. The gut was imaged under the microscope (Olympus SZX7, Japan) with a camera system MZX81 (MshOt, China) after 2 h. This method typically produces very clear results—either a “Yes” or “No” outcome. In simpler terms, the signal will either be present or absent in a specific region of the gut. The presence of the signal in the anterior and posterior midgut, or its absence, was recorded accordingly. *In vivo* measurement of countercurrent flow was performed as previously described [23]. However, due to the gut’s absorption of the dye in *B. dorsalis*, the dye accumulation anterior midgut might be a combined result of gut absorption and countercurrent flow pushback.

### Monitor of intestinal peristalsis frequency

Adult female *B. dorsalis*, aged between five and eight days, underwent a 2-h starvation period in a plastic container maintained at 28°C. The flies were then infected with infection solution supplied with FITC dextran (Sigma, Germany). The flies were placed on a sticky tape with their backs fixed to the tape with their abdomen facing upside. One minute gut peristalsis videos were recorded using the microscope (Olympus SZX7, Japan) with a camera system MZX81 (MshOt, China) 2 h after infection. The gut peristalsis times were counted from the videos.

### Gut reactive oxygen species staining and quantification

To quantify ROS using dihydroethidium (DHE), flies were chilled on ice to induce anesthesia, followed by dissection of their guts in Schneider’s insect medium. The dissected guts were then immediately submerged in a 30 mM solution of DHE (Life Technologies) and incubated for 7 min at ambient temperature. Afterward, the guts were rinsed twice with Schneider’s insect medium and promptly visualized using a SP8 LIGHTNING confocal microscope manufactured by Leica (Germany). The signal intensity was quantified using FIJI.

Rhodamine 6G (R6G) HOCI sensor was purchased from Heliosense (Xiamen, China). To facilitate microscope examination of the R6G fluorescence signal, both the infection solution and the sucrose control solution were supplemented with 50 μM of R6G. The flies were permitted to consume these solutions for a period of 2 h. Following a 2 h interval post infection, the flies were gathered and dissected in Schneider’s insect medium. The dissected guts were then mounted in an antifade medium sourced from Beyotime (China), and promptly inspected using a SP8 LIGHTNING confocal microscope.

For detecting H_2_O_2_ in the gut, we used hydrogen peroxide assay kit (Beyotime, China) following the manufactural protocol. Briefly, flies were anesthetized on the ice and the guts were dissected in PBS at desired time points. The guts were collected and homogenized with glass beads. The kit is based on the production of trivalent iron ions by oxidizing divalent iron ions with hydrogen peroxide, which then forms a purple product with xylenol orange. The absorbance was measured at 560 nm. H_2_O_2_ concentration was adjusted to the protein quantity using bicinchoninic acid assay Protein Assay Kit (Beyotime, China).

### Ribonucleic acid interference

Primers bearing the T7 ribonucleic acid (RNA) polymerase promoter sequence (5′- GGATCCTAATACGACTCACTATAGG-3′) at their 5′ ends were utilized for cloning the desired sequence fragments via polymerase chain reaction (PCR). The primer sequences are detailed in Supplementary Table 1. A quantity of 1 μg of the PCR product served as the template for in vitro synthesis of double-stranded RNA (dsRNA) using the T7 Ribomax Express RNAi System from Promega (Madison, WI, USA). The concentration of the synthesized dsRNA was measured at 280 nm using a NanoDrop 2000 Spectrophotometer (Thermo Fisher Scientific Inc.). The integrity and quality of the dsRNA were assessed through agarose gel electrophoresis. Needles for dsRNA injection were fabricated using a puller (PC 10, Narishige, Tokyo, Japan) set at a heat level of 60.8. The Nanoinject III system from Drummond Scientific (USA) was employed for microinjecting the dsRNA. For the RNAi experiments, 1 μl of a 2 μg per microliter dsRNA solution was injected into the thorax of flies aged 5 to 8 days. The experiments were conducted three days post injection. As a control, flies injected with *ds-egfp* were used.

### Ribonucleic acid extraction and quantitative polymerase chain reaction

To analyze gene expression, RNA was extracted from either 8 whole flies or 20 guts. Subsequently, 500 nanograms of total RNA or equivalent tissue samples were reverse transcribed using PrimeScript RT enzyme from TAKARA, in conjunction with a blend of oligo-dT and random hexamer primers. Quantitative PCR (qPCR) reactions were carried out on complementary deoxyribonucleic acid (cDNA) samples in a 20 μl volume, comprising 10 μl of SYBR Green Mix (Vazyme, China), 400 nM of each primer, and 2 μl of cDNA (diluted 1:10). These reactions were conducted on an FQD-96X instrument (Bioer, China), utilizing 96 well plates. The PCR protocol involved an initial denaturation step at 95°C for 3 min, followed by 40 cycles of 95°C for 15 s and 60°C for 30 s. Relative gene expression levels were determined using the 2^-ΔΔCt^ method. A minimum of three independent biological replicates were performed for each experiment. RpL32 served as the reference gene. The primers utilized in the qPCR analysis are listed in Supplementary Table 1.

### Hemolymph extraction and tyramine detection

For collecting hemolymph, eight individuals were placed in a bottom opened 0.6 ml tube covered with glass beads. The tube was transferred into a 1.5 ml tube and spun in a centrifuge for 10 min at 4°C with a rotational speed of 1500 rpm. To clear hemolymph samples, an additional centrifugation step was carried out for 5 min at 13 000 rpm. For gut and Malpighian tubules tyramine detection, the desired tissues were dissected in PBS. The whole flies, gut, Malpighian tubules and hemolymph samples were homogenized in PBS. For bacteria tyramine detection, overnight culture (OD600 = 100) was used. Tyramine detection was performed using insect tyramine kit (MEMIAN, China) according to the manufactural protocol with 10 μl infection solution. All data were normalized to a single fly. To normalize the tyramine content of ingested bacteria, we measured bacterial consumption by individual flies. Twenty flies were placed in a plastic box containing 500 μl of infection solution, and the remaining volume was measured at 0 h (see Supplementary Fig. 1B). The tyramine content consumed per fly was then calculated.

### Bacteria deoxyribonucleic acid extraction and 16S ribosomal ribonucleic acid amplicon sequencing

Bacteria DNA extraction and 16S ribosomal RNA (rRNA) amplicon sequencing were performed using the established method in the lab. DNA was extracted from the guts of 40 flies aged 5–8 days after emergence for each biological replicate utilizing the E.Z.N.A. Soil DNA kit provided by Omega (Norcross, GA, USA), following the manufacturer’s guidelines. Two or three biological replicates were carried out for consistency. To amplify the 16S rRNA gene’s variable regions V3 and V4, the broad-spectrum forward primer 341F (CCTAYGGGRBGCASCAG) and reverse primer 806R (GGACTACNNGGGTATCTAAT) were employed in conjunction with Phusion High Fidelity PCR Master Mix from New England Biolabs (Beverley, MA). The PCR amplification protocol involved an initial heating at 95°C for 5 min, followed by 35 cycles of annealing at 56°C for 45 s, extension at 72°C for 1 min, and denaturation at 94°C for 45 s, culminating in a final extension at 72°C for 10 min. The PCR products were then submitted to Novogene’s Experimental Department for sequencing. Upon receiving the raw sequencing data, the single end reads were assigned to their respective samples based on unique barcodes and truncated by removing the barcode and primer sequences. Quality filtering was applied to the raw reads under specified conditions to generate high quality clean reads, utilizing the Cutadapt (version b1.9.1, accessible at http://cutadapt.readthedocs.io/en/stable/) quality control process as described by Martin in 2011. The reads were then compared against the reference database (Silva database, accessible at https://www.arb-silva.de/) using the UCHIME algorithm (available at http://www.drive5.com/usearch/manual/uchime_algo.html) to detect and subsequently remove chimeric sequences, yielding the final set of clean reads. Alpha diversity (Simpson index) was calculated using qime and visualized with R software.

### Statistical analysis

Each experiment was independently replicated at least three times, with error bars showing standard deviation. Statistical analysis was performed using appropriate tests in GraphPad Prism, and *P* values were denoted as follows: ns (*P* ≥ .05), ^*^ (0.01 < *P* < .05), ^**^ (0.001 < *P* ≤ .01), ^***^ (0.0001 < *P* ≤ .001), and ^****^ (*P* ≤ .0001). *P* values were listed in Supplementary Table 2.

## Results

### Pathobiont *Providencia rettgeri* triggers a countercurrent flow in *Bactrocera dorsalis*

*P. rettgeri* is a Gram-negative bacteria that can be found in *B. dorsalis* and other Tephritidae fruit flies gut microbiota in both lab and wild populations [28–33]. Our results and others have shown that *P. rettgeri* may benefit host fitness under certain circumstances [29, 32, 34]. However, *P. rettgeri* is also a pathogenic bacteria to many insects, suggesting that *P. rettgeri* could be a pathobiont of *B. dorsalis* [30, 35–37]. Our previous research has shown *P. rettgeri* promotes *B. dorsalis* larval growth but is also a potent inducer for host immune response [34, 37]. We confirmed that oral infection with our lab strain *P. rettgeri* led to host death, suggesting that *P. rettgeri* is a pathobiont in *B. dorsalis* adults (Supplementary Fig. 1A).

**Figure 1:**
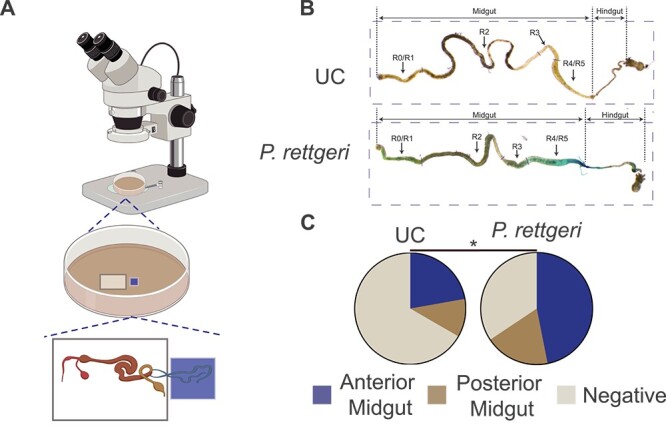
Pathobiont *P. rettgeri* triggers a countercurrent flow in *B. dorsalis.* (A) Schematic diagram of *ex vivo* intestinal countercurrent detection method. Two adjacent wells in the PDMS plate were filled with cell culture. The connected gut and Malpighian tubules were put into two adjacent wells, respectively, and the Malpighian tubules well was supplemented with 0.05% bright blue solution. A drop of mineral oil was added to the junction of the two wells to cover the tubules preventing tissue dry out. (B and C) *P. rettgeri* gut infection promotes gut countercurrent flow. Oral infection induced a strong Malpighian tubules–gut countercurrent flow indicated by the accumulation of blue dye in the anterior midgut (B) and quantification in (C) (*n* = 36 for UC, *n* = 32 for *P. rettgeri*). UC: unchallenged. ^*^P value <.05, chi-square test, see detailed *P* values in Supplementary Table 2.

We have recently shown that pathogenic bacteria oral infection activates a renal–gut countercurrent flow in *D. melanogaster* that plays an important role in gut epithelial renewal [27]*.* To test if pathobiont can induce countercurrent flow, we set up an *ex vivo* assay to test the existence of countercurrent flow in *B. dorsalis*. Briefly, flies were subjected to either oral infection for 2 h with *P. rettgeri,* or a control sucrose solution, then switched to fresh fly food (Supplementary Fig. 1B). The experiments were performed as previously described [23] (Fig. 1A). The results suggested that *P. rettgeri* infection induced the rapid Malpighian tubules absorption of Brilliant blue dye 2 h post infection. We observed the dye moved forward to the midgut and accumulated along the gut, including the anterior midgut and posterior midgut (Fig. 1B). We noticed an increase in the percentage of the gut displaying dye from 33.3%–65.6% upon oral infection. Almost half of the gut has a strong dye signal in the anterior part, suggesting a more robust flow than the unchallenged condition (Fig. 1C). Moreover, we also saw the countercurrent flow positive gut in the unchallenged condition, suggesting this flow might have a role with gut resident bacteria (Fig. 1C). We also performed an *in vivo* countercurrent flow measurement experiment. The results also suggested a stronger countercurrent flow following *P. rettgeri* infection (Supplementary Fig. 1C). We also examined whether the intensity of countercurrent flow was determined by different dye absorption by Malpighian tubules. The results showed that the Malpighian tubules had similar dye accumulation suggesting a similar Malpighian tubules excretion ability (Supplementary Fig. 1D). Altogether, these data revealed the existence of a Malpighian tubules–gut countercurrent flow after pathobiont oral infection in *B. dorsalis*.

### Countercurrent flow in *Bactrocera dorsalis* requires the aquaporin Prip and promotes early stage bacteria clearance

**Figure 2:**
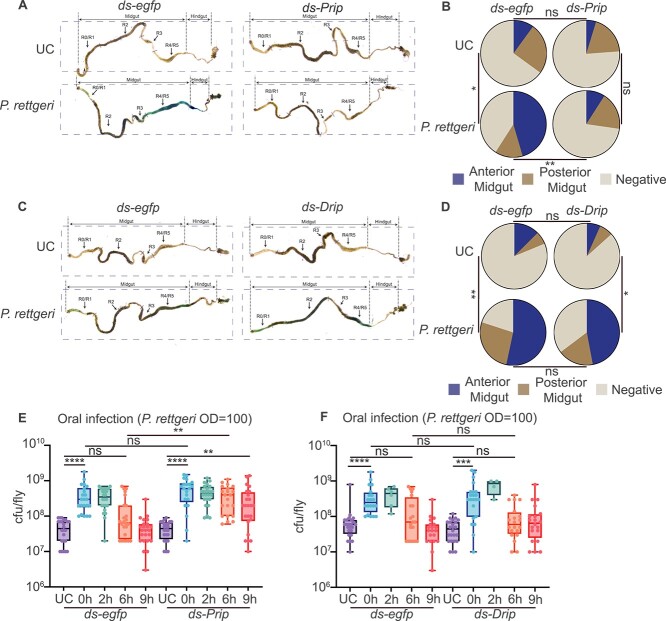
Countercurrent flow in *B. dorsalis* requires the aquaporin Prip and promotes early stage bacteria clearance. (A and B) *Prip* is required for gut countercurrent flow generation. *Prip* RNAi flies showed very weak blue dye accumulation in the anterior midgut (A) and quantification in (B) (*n* = 20–33). *egfp* RNAi flies were used as control. UC: unchallenged. (C and D) *Drip* RNAi did not affect gut countercurrent flow generation. There was no significant difference regarding blue dye accumulation in the gut between *drip* RNAi and *egfp* RNAi flies indicated by countercurrent flow assay (C) and quantification in (D). *egfp* RNAi flies were used as control (*n* = 15–17). (E) *Prip* RNAi decreased the flies’ ability to eliminate gut invading bacteria. *egfp* RNAi and *Prip* RNAi flies showed significant cfu counts differences at 6 h after the infection. *Prip* RNAi flies could not eliminate invading bacteria at 9 h after infection. *egfp* RNAi flies were used as control. Each data point represents bacteria burden from a single fly (*n* = 21–24). UC: unchallenged. (F) *Drip* RNAi did not affect bacteria elimination. *Drip* RNAi did not affect the flies’ ability to eliminate gut-invading bacteria. *egfp* RNAi flies were used as control. Each data point represents bacteria burden from a single fly (*n* = 4–24). UC: unchallenged. ^*^P value <.05; ^**^P value <.01; ^***^P value <.001; ^****^P value <.0001; ns non-significant. B and D were analyzed using the chi-square test, E and F were analyzed using the Kruskal–Wallis test with post-hoc Dunn’s test; see detailed *P* values in Supplementary Table 2.

Aquaporins are a family of membrane water channels, some of which are also capable of transporting glycerol [38]. In *D. melanogaster*, aquaporin Drip is required in Malpighian tubules stellate cells, which are responsible for regulating ion balance and fluid secretion, for countercurrent flow formation [23, 39]. We then explored the role of aquaporins in controlling Malpighian tubules–gut countercurrent flow in *B. dorsalis*. We first confirmed the aquaporin *Prip* and *Drip* are expressed in the Malpighian tubules (Supplementary Fig. 2A and B). We knocked down *Prip* and *Drip* using RNAi by injecting dsRNAs (Supplementary Fig. 2D and E). The results showed that *Prip* RNAi flies failed to generate a strong countercurrent flow after *P. rettgeri* infection as indicated by the absence of the dye in the anterior midgut, suggesting that Malpighian tubules–gut countercurrent flow formation requires Prip (Fig. 2A and B). However, we saw no difference in dye accumulation between the *Drip* RNAi and the control groups, indicated by the accumulation of the dye in the anterior midgut, suggesting *Drip* may not be necessary for countercurrent flow formation (Fig. 2C and D). Above results suggested a role of *Prip* but not *Drip* in countercurrent flow formation in *B. dorsalis*.

The observation that counterflow is induced upon pathobiont infection points to its possible role in host defense. Next, we investigated whether this Malpighian tubules–gut retro flow is required for early stage bacteria clearance. Both *Prip* and *Drip* RNAi flies and control *egfp* RNAi flies were fed with *P. rettgeri* for 2 h and then switched to fresh food. We monitored bacteria persistence in the gut for the next 9 h. Importantly, we found that *Prip* RNAi flies lost their ability to clear invading bacteria (Fig. 2E). However, *Drip* RNAi flies, which have normal retro-flow, have successfully cleared invading bacteria 6 h after *P. rettgeri* infection, showing no difference compared to the control group (Fig. 2F). As Prip but not Drip is involved in the countercurrent flow formation, our results points to an important role of Malpighian tubules–gut retro-flow in bacterial clearance. In conclusion, the above results suggested that the aquaporin Malpighian tubules–gut countercurrent flow which involved Prip is required for efficient bacteria clearance after oral infection in *B. dorsalis*.

### Exogenous tyramine is a signaling molecule that triggers Malpighian tubules–gut countercurrent flow

**Figure 3:**
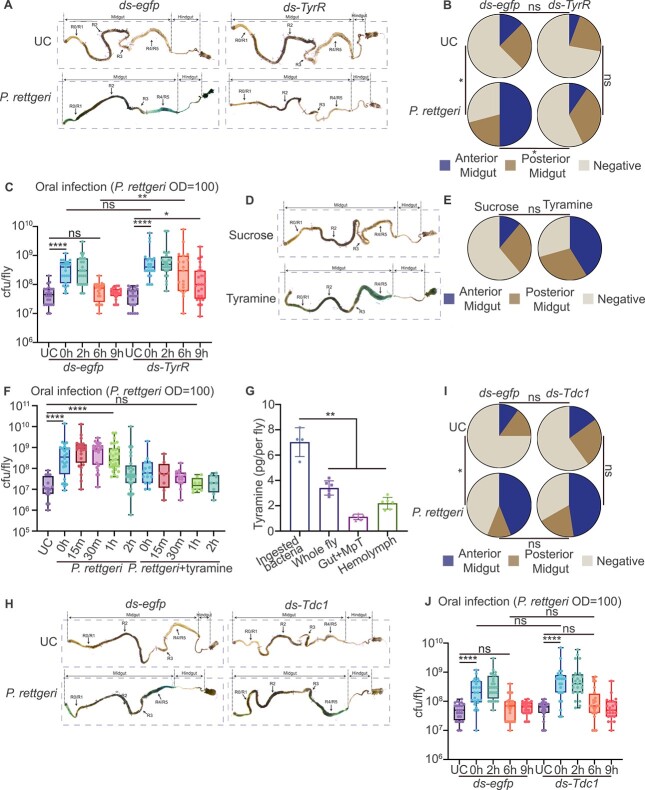
Exogenous tyramine is a signaling molecule that triggers Malpighian tubules–gut countercurrent flow. (A and B) *TyrR* RNAi reduces Malpighian tubules–gut countercurrent. *TyrR* RNAi flies showed very weak blue dye accumulation in the anterior midgut (A) and quantification in (B). *egfp* RNAi flies were used as control (*n* = 18–24). UC: unchallenged. (C) *TyrR* RNAi decreased the flies’ ability to eliminate gut invading bacteria. The Cfu counts in *TyrR* RNAi flies were significantly higher at 6 h compared to the control group. *egfp* RNAi flies were used as control. Each data point represents bacteria burden from a single fly (C) (*n* = 19–24). UC: unchallenged. (D–F) Tyramine promotes Malpighian tubules–gut countercurrent flow and bacteria clearance. Tyramine-fed flies showed a strong accumulation of bright blue dye in the anterior midgut (D) and quantification in (E) (*n* = 18 for sucrose, *n* = 17 for tyramine). Co-fed tyramine and bacteria promoted the bacterial elimination (F) (*n* = 8–24). Each data point represents bacteria burden from a single fly (F). UC: unchallenged. (G) The tyramine content of ingested *P. rettgeri* and various tissues of *B. dorsalis* of a single fly. *P. rettgeri* produced higher amount of tyramine compared to fly tissues. Each datapoint represents the tyramine content from a single fly (*n* = 4–6). (H–J) endogenous tyramine did not affect Malpighian tubules–gut countercurrent flow and bacteria elimination. Silencing *Tdc1* did not affect the formation of Malpighian tubules–gut countercurrent flow (H) and quantification in (I). *Tdc1* RNAi did not affect the clearance of *P. rettgeri* in the gut of *B. dorsalis* (J). *egfp* RNAi flies were used as control [*n* = 20–25 for (H), *n* = 23–24 for (J)]. Each data point represents bacteria burden from a single fly (J). UC: unchallenged. ^*^P value <.05; ^**^P value <.01; ^****^P value <.0001; ns non-significant. B, E, and I were analyzed using chi-square, C, F, and J were analyzed using Kruskal–Wallis test with post-hoc Dunn’s test, G was analyzed by Mann–Whitney U test, see detailed *P* values in Supplementary Table 2.

The biogenic amine tyramine regulates many aspects of animal physiology. Tyramine also controls Malpighian tubules function in insects [40, 41]. We hypothesized that tyramine might be the signaling molecule inducing the countercurrent flow from the Malpighian tubules to the gut. To test this hypothesis, we first knocked down *TyrR* in *B. dorsalis* (Supplementary Fig. 3A). TyrR is the tyramine receptor regulating Malpighian tubules stellate cell activity (Supplementary Fig. 2C) [42]. We monitored Malpighian tubules–gut countercurrent flow using in vitro countercurrent experiments. *TyrR* knockdown strongly decreased the intensity of this retro-flow, as the dye could not reach the anterior midgut in *TyrR* RNAi flies after infection (Fig. 3A and B). More importantly, *TyrR* RNAi flies also had a weaker ability to clear invading bacteria (Fig. 3C). These data indicated that tyramine contributes to the Malpighian tubules–gut countercurrent flow. To further strengthen our conclusions, we directly fed *B. dorsalis* with tyramine and detected countercurrent flow. The results showed that feeding flies with a diet supplemented with tyramine enhanced gut countercurrent flow, promoting dye accumulation in the anterior midgut, similar to the oral infection (Fig. 3D and E). Furthermore, feeding tyramine significantly increased flies’ ability to eliminate invading bacteria. Tyramine-fed flies eliminated invading bacteria within 1 h after infection compared with 2 h in the control flies (Fig. 3F). We further tested whether TyrR signaling could regulate Prip’s transcriptional activity. The results showed that knocking down TyrR had no significant impact on *Prip* expression, suggesting that TyrR signaling may not directly regulate aquaporin Prip activity (Supplementary Fig. 3B). Moreover, tyramine feeding did not rescue the countercurrent flow phenotype in *Prip* RNAi flies, indicating that aquaporin might act downstream of TyrR signaling (Supplementary Fig. 3C).

It is well characterized that bacteria, for example, *Providencia* bacteria, can produce large amounts of tyramine regulating host physiology [43]. We wondered if the tyramine was produced by the flies or if the bacteria secret it. We first examined the tyramine quantity in ingested *P. rettgeri* and *B. dorsalis*. The results showed that both ingested *P. rettgeri* and *B. dorsalis* could produce tyramine (Fig. 3G). Ingested *P. rettgeri* produces higher amount of tyramine than the flies, suggesting a possible role of bacteria-derived tyramine in regulating Malpighian tubules functions. To validate our assumption, we knocked down the tyrosine decarboxylase 1 (*Tdc1*) in *B. dorsalis* (Supplementary Fig. 3D). Tdc1 is the enzyme that catalyzes the decarboxylation of tyrosine to tyramine and is necessary for renal function regulation in *D. melanogaster*. The results showed that *Tdc1* RNAi flies had a similar Malpighian tubules–gut countercurrent flow as *egfp* RNAi flies, and bright blue dye could reach the gut R2 region after *P. rettgeri* infection (Fig. 3H and I), suggesting that intrinsic tyramine synthesis pathway is not required for countercurrent flow formation. Consistent with this finding, we also found that the bacteria clearance ability remained unchanged in *Tdc1* RNAi flies (Fig. 3J). To further strengthen the conclusion, we detected tyramine levels in the gut and hemolymph in *Tdc1* RNAi flies after *P. rettgeri* infection. The results showed that *Tdc1* RNAi did not affect tyramine levels in the gut and hemolymph compared with the control group (Supplementary Fig. 3F and G). In both tissues, we still observed an increase in tyramine content in both gut and hemolymph, suggesting that these tyramines are exogenous. Another tyrosine decarboxylase gene, Tdc2, has been identified in *B. dorsalis*. We attempted to knock down *Tdc2* and evaluated its role in countercurrent formation and bacterial elimination. However, our results indicated that *Tdc2* RNAi did not significantly affect either retro-flow formation or bacterial clearance (Supplementary Fig. 3H and 3I). Taken together, these data suggested that tyramine secreted by pathogenic bacteria activates the countercurrent flow.

### Duox is required for early stage *Providencia rettgeri* clearance

**Figure 4:**
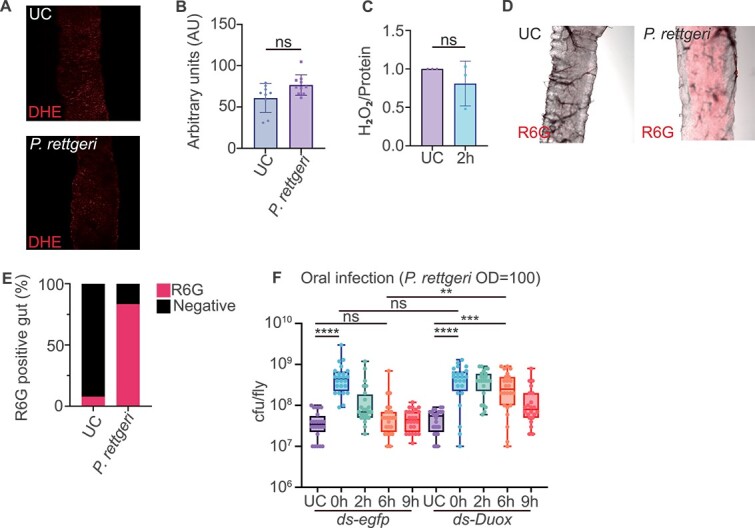
Duox is required for early stage *P. rettgeri* clearance. (A and B) superoxide content in the gut after *P. rettgeri* gut infection. Superoxide content in the gut was revealed by DHE staining (A) and quantification in (B). The data represents the average of 3 biological replicates. Each data point represents DHE signal intensity from a single fly gut (R2 region; *n* = 9 for UC and *n* = 10 for *P. rettgeri*). UC: unchallenged. (C) *P. rettgeri* gut infection did not activate gut H_2_O_2_ production. The H_2_O_2_ content was normalized to the UC group, which was set as 1.Each data point represents H_2_O_2_ content from a pooled sample of 10 flies. UC: unchallenged. (D and E) HOCl staining in the gut after *P. rettgeri* infection. HOCl sensor R6G staining showed an increased amount of ClO^−^ in the gut after infection (D). The percentage of R6G-positive guts increased after infection (E). The data represents the results of 3 biological replicates (*n* = 13 for UC, *n* = 18 for *P. rettgeri*). UC: unchallenged. (F) *Duox* RNAi decreased the flies’ ability to eliminate gut invading bacteria. Removing ROS production by *Duox* RNAi delayed bacteria elimination. *egfp* RNAi flies were used as control. Each data point represents bacteria burden from a single fly (*n* = 21–24). UC: unchallenged. ^**^P value <.01; ^***^P value <.001; ^****^P value <.0001; ns non-significant. B and C were analyzed using Mann–Whitney U test. F was analyzed Kruskal–Wallis test with post-hoc Dunn’s test, see detailed *P* values in Supplementary Table 2.

Gut pathobiontic bacteria can activate host Duox–ROS production to maintain gut homeostasis in *D. melanogaster* [6]. We explored the role of the Duox–ROS production system in *P. rettgeri* clearance. We first checked ROS production in the gut after *P. rettgeri* infection 2 h post infection in *B. dorsalis*. The primary Duox ROS product is HOCl, while superoxide (O_2_^−^) and H_2_O_2_ are intermediate products [18]. We monitored each of these ROS in the gut using appropriate assays: DHE for superoxide, hydrogen peroxide assay kit for H_2_O_2_ and the R6G HOCI sensor for HOCl. We did not detect any increase in superoxide production (Fig. 4A and B), nor did we see an increased amount of H_2_O_2_ (Fig. 4C). However, we saw a strong HOCl burst in the gut region corresponding to region R2 at 2 h post-infection (Fig. 4D and E, Supplementary Fig. 4A) [44]. To check whether ROS is necessary for bacteria clearance, we monitored bacteria persistence in *Duox* RNAi flies (Supplementary Fig. 4B). We found that *Duox* RNAi flies had a weaker ability to clear invading bacteria as these flies cannot remove the bacteria until 9 h after infection, suggesting that Duox-mediated ROS production mechanism is crucial for early *B. dorsalis* gut immune response (Fig. 4F). To further support our conclusion, we co-fed the flies with bacteria and vitamin C, a potent antioxidant. We found that *B. dorsalis* cannot clear *P. rettgeri* even at 9 h after infection when we remove the ROS using vitamin C (Supplementary Fig. 4C). Altogether, our data showed that *B. dorsalis* relied on Duox–ROS to remove excessive *P. rettgeri* at the early stage of infection.

### Countercurrent flow ensures *Providencia rettgeri* clearance by promoting reactive oxygen species accumulation and gut peristalsis

**Figure 5:**
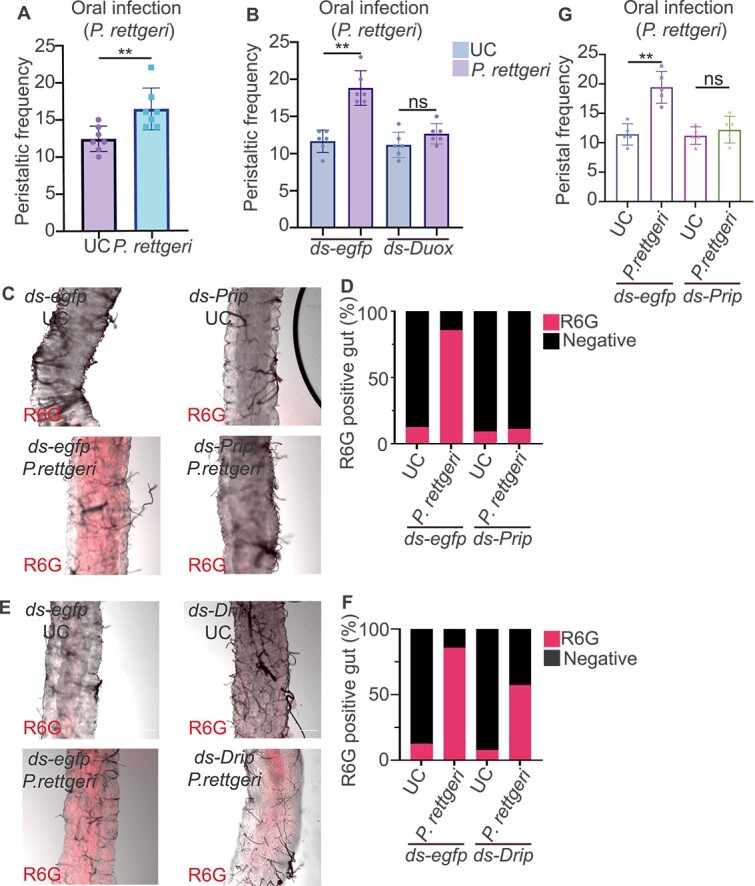
Countercurrent flow ensures *P. rettgeri* clearance by promoting ROS accumulation and gut peristalsis. (A) The intestinal peristalsis frequency increased after *P. rettgeri* gut infection. Two hours after *P. rettgeri* gut infection, the frequency of intestinal peristalsis increased significantly. Each data point represents data of a single fly (*n* = 7). UC: unchallenged. (B) *Duox* RNAi decreases the intestinal peristalsis frequency after *P. rettgeri* gut infection. Two hours after *P. rettgeri* gut infection, the frequency of intestinal peristalsis did not increase in *Duox* RNAi flies. Each data point represents data of a single fly (*n* = 6). UC: unchallenged. (C and D) *Prip* RNAi reduced the ClO^−^ accumulation in the gut. Only *egfp* RNAi flies showed a strong gut R6G signal after *P. rettgeri* oral infection (C) and quantification in (D). *egfp* RNAi flies were used as control (*n* = 9–16). UC: unchallenged. (E and F) *drip* RNAi did not affect the ClO^−^ accumulation in the gut. Both *egfp* RNAi and *drip* RNAi flies showed a strong R6G fluorescence signal in the gut after *P. rettgeri* oral infection (E) and quantification in (F). *egfp* RNAi flies were used as control (n = 13–16). UC: unchallenged. (G) *Prip* RNAi reduces the gut peristalsis frequency. *Prip* RNAi flies did not increase gut peristalisis after infection. *egfp* RNAi flies were used as control. Each data point represents data of a single fly *(n* = 5). UC: unchallenged. ^**^P value <.01; ns non-significant. A, B, and G were analyzed using Mann–Whitney U test; see detailed *P* values in Supplementary Table 2.

Recent studies have highlighted a possible key role of Duox in bacterial clearance by promoting muscle contraction [16, 17]. We therefore investigated whether peristalsis also contribute to *B. dorsalis* ability to eliminate bacteria. We fed flies with FITC-labeled dextran and monitored gut peristalsis upon oral infection with the Gram-negative bacteria *P. rettgeri* infection. We observed that oral infection increased gut peristalsis by 32% (Fig. 5A). This effect was absent in *Duox* RNAi flies indicating that gut peristalsis requires by Duox in *B. dorsalis* (Fig. 5B). Consistent with this finding, co-fed the flies *P. rettgeri* with vitamin C also inhibited increased gut peristalsis (Supplementary Fig. 5A).

We wondered whether blocking Malpighian tubules–gut countercurrent flow would affect the ROS amount and gut peristalsis. We quantified the HOCl amount in *Prip* RNAi flies. The results showed that *Prip* RNAi strongly decreased the number of HOCl-positive flies after oral infection, as indicated by the R6G HOCl sensor (Fig. 5C and 5D). This result suggested that countercurrent flow may promote gut bacteria clearance through HOCl distribution along the gut. To further support our conclusion, we also quantified HOCl amount in *Drip* RNAi flies. *Drip* RNAi did not have a strong impact on the number of HOCl-positive flies (Fig. 5E and F). To exclude the possibility that *Prip* RNAi would directly affect AMPs and ROS production, we examined *Diptericin* and *Duox* expression in *Prip* RNAi flies. The RT-qPCR analysis showed that *Prip* RNAi did not affect the *Diptericin* and *Duox* expression (Supplementary Fig. 5B and C). Because Duox played an important role in gut muscle contraction and food defecation, we checked gut peristalsis in *Prip* RNAi flies. *Prip* RNAi inhibited the gut peristalsis increase after infection (Fig. 5G). Taken together, these observations suggested that the Malpighian tubules–gut countercurrent flow ensures gut pathobiont bacteria clearance by promoting ROS-mediated gut peristalsis.

### Gut symbiont promotes countercurrent flow but not reactive oxygen species production

**Figure 6:**
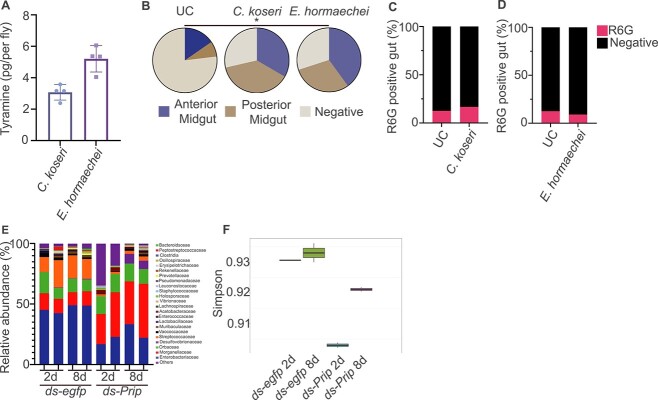
Gut symbiont promotes countercurrent flow but not ROS production. (A) The tyramine content in ingested symbiotic bacteria. Each datapoint represents the tyramine content from a single fly (*n* = 6). (B) Symbiotic *C. koseri* and *E. hormaechei* gut infection promote gut countercurrent flow (*n* = 13–16). ^*^P value <.05. Chi-square test, see detailed *P* values in Supplementary Table 2. (C and D) HOCl burst *C. koseri* and *E. hormaechei* infected flies *(n* = 22–24). (E and F) *Prip* RNAi altered gut microbiota composition in *B. doralis*. Relative genus-level abundance profiles of bacteria in the gut (E). Gut microbiota community diversity was measured using the Simpson index (F). *egfp* RNAi flies were used as control (*n* = 2).

We wondered whether gut symbionts can also release tyramine and promote the formation of countercurrent flow. *C. koseri* and *E. hormaechei* are dominant microbiota species in *B. dorsalis* [28]. We found these two bacteria also produce a considerable amount of tyramine (Fig. 6A, compared with Fig. 3G). We expect these bacteria can also induce countercurrent flow. By in vitro assay, we found that *C. koseri* and *E. hormaechei* can induce a stronger countercurrent flow (Fig. 6B). This result suggests that symbionts also contribute to Malpighian tubules–gut countercurrent flow formation. We have shown earlier that countercurrent flow ensures bacteria clearance by promoting pathobionts-induced ROS-mediated gut peristalsis. So we tested whether symbiont also contribute to this process. We monitored HOCl production in the flies infected with *C. koseri* and *E. hormaechei*. The results showed that neither *C. koseri* nor *E. hormaechei* induce a strong HOCl burst at the early stage of infection (Fig. 6C and D).

We next examined whether countercurrent flow is also required for microbiota control. We performed 16S rRNA sequencing in *Prip* RNAi flies to validate this assumption. Regarding relative abundance, the high-throughput sequencing revealed that *Enterobacteriaceae*, *Morganellaceae*, *Orbaceae*, and *Streptococcaceae* dominated the gut regions of *egfp* RNAi control flies. However, *Enterobacteriaceae*, the most abundant bacteria species, decreased to 22.5% in *Prip* RNAi flies compared to the control group. The less dominant species *Streptococcaceae* reduced to very low levels, becoming a minor microbiota species in the flies’ gut. On the contrary, we found an increase in *Morganellaceae*, which accounts for around 35.4% of the total microbiota in *Prip* RNAi flies. One minor species in control flies, *Desuifovobrionaceae*, also became a dominant microbiota species in *Prip* RNAi flies (Fig. 6E). These results suggested that inhibiting Malpighian tubules–gut countercurrent flow by *Prip* knockdown caused microbiota dysplasia in the gut. Furthermore, gut microbiota species richness and evenness were reduced in the *Prip* RNAi flies, as indicated by Simpson diversity indices (Fig. 6F).

## Discussion

In this study, we identified a physiological mechanism essential for microbiota homeostasis contributed by pathobiont and symbiotic bacteria. We demonstrated that the host generates a Malpighian tubules–gut countercurrent flow in response to the presence of pathobiont and symbiotic bacteria-derived tyramine. This flow facilitates the accumulation of ROS upon recognition of pathobiont, and in turn, ROS ensures proper gut peristalsis. We also provided evidence that this flow is involved in maintaining microbiota homeostasis. This work suggested a mechanism involving both pathobiont and symbiotic bacteria that promotes the accumulation of ROS. Furthermore, our work also proved the involvement of Malpighian tubules in gut microbiota homeostasis, in addition to their well-known functions in regulating excretion physiology.

Our results suggested that both pathobionts and symbionts contribute to the formation of Malpighian tubules–gut countercurrent flow, while ROS is produced by pathobionts in the early stage of infection. By interaction with both pathobionts and symbionts, host developed a successful gut immune response which is crucial for microbiota control. Growing evidence has shown the complexity of pathobionts and symbionts interactions. For example, it is common for pathobionts to adopt various mechanisms to promote their fitness. In cotton leafworm *Spodoptera littoralis*, extracellular symbiont *Enterococcus mundtii* produces potent antimicrobials selectively limiting pathobiont expansion, providing protection against infection [45]. This phenomenon is also observed in many other insects [46, 47]. These examples highlight the possibility that interactions between microbes could be a key factor in maintaining microbe-host homeostasis. Here, we provided a new model that both pathobiont and gut symbiont shape the host gut immune response. Similar to previous findings, we found that pathobionts but not symbionts can induce host ROS production and gut peristalsis, implying host can retain most of the beneficial symbionts. It is well known that pathogenic bacteria, such as *Pectobacterium carotovorum* subsp. *Carotovorum* (*Ecc15*) and others, release significant amounts of uracil, which is detected by an as-yet unidentified host receptor [6]. Host then activated Duox activity upon recognition of these uracil [20, 48, 49]. However, data indicate that gut-beneficial symbionts, such as *Commensalibacter intestini* A911T, *Acetobacter pomorum*, and *Lactobacillus plantarum*, do not release uracil, and therefore, do not activate Duox or trigger a HOCl burst [6]. *Gluconobacter morbifer* G707T, a minor member of the natural gut-dwelling pathobiont community, can activate Duox through uracil release [6]. Although untested, we hypothesize that *C. koseri* and *E. hormaechei*, which we isolated from *B. dorsalis*, are beneficial microbes that do not produce uracil, whereas *P. rettgeri* might do so. We used a short feeding scheme to investigate the host immune response in the early stage of infection. It is possible that under continuous infection or long-term colonization, a different mechanism could exist in insects. An open question remains as to whether pathobionts and symbionts might encounter a similar challenge in relation to gut peristalsis. The central question here is how gut microbes successfully establish a stable association with the host’s digestive tract. This process involves multiple mechanisms, including gut peristalsis, biofilm formation, and inter/intra-species competition. Future works are required to understand how pathobionts and symbionts might respond to host immunity distinctly.

The fact that ROS is well preserved by the Malpighian tubules–gut countercurrent flow proved that insects own a delicate system to prevent the loss of valuable resources. The countercurrent flow from Malpighian tubules to the anterior gut in *B. dorsalis* appears to be conserved in other insects. Recently, we found that countercurrent flow induced by *Ecc15* infection was crucial for gut epithelial renewal in *D. melanogaster* [27]. Early reports in locusts showed that a counter-current flow from Malpighian tubules occurred when the locusts were starved [25]. By using dye feeding and observation of its distribution in the gut, Terra and colleagues proposed the existence of countercurrent flow in other insects, including Lepidoptera [50], Coleoptera [51], and Orthoptera [52]. Our results suggested aquaporins’ critical role in forming the countercurrent flow in *B. dorsalis*. Aquaporins are also enriched in the anterior midgut in *D. melanogaster*, implying that the anterior midgut might be a major region for water absorption, thus forming a complete water flow cycle consisting of Malpighian tubules, hemolymph, and the gut [44, 53]. In the absence of tissue-specific knockdown tools, our results do not identify the specific tissue in which *Prip* or *TyrR* is required. It remains possible that these key genes are essential in tissues other than the Malpighian tubules. Moreover, although *Prip* RNAi does not have a strong impact on Duox and IMD target genes expression, it may exert its effects through other pathways.

The originally proposed function of countercurrent flow is the recycling of enzymes to increase digestion efficiency. The most solid evidence is that the enzyme concentration is higher within the ectoperitrophic space than in the endoperitrophic space [26]. Our previous results suggested that this countercurrent flow could bring renal-derived Upd3 to regulate gut renewal upon oral infection [27]. Here, we found that the retro-flow also participated in the immune response by promoting ROS accumulation and gut peristalsis. It is reasonable to expect that this mechanism could also promote the accumulation of other antimicrobial agents, such as AMPs, or regulate the immune-metabolism relationship. Our results suggest that this process may also play a role in regulating microbiota homeostasis. *Prip* RNAi flies exhibited a clear trend of microbiota dysplasia. However, no significant differences in countercurrent flow were observed between *Prip* RNAi flies and control flies without additional infection. This may be due to the need for more precise methods to monitor fluid flow within the ectoperitrophic space, rather than relying solely on dye as an indicator.

The generation of ROS is a highly conserved physiological response to microbial presence across all organisms [19]. It is well-established that Duox plays a key role in microbiota regulation. Evidence from mosquitoes, *B. dorsalis*, and other insects underscores the importance of Duox in maintaining proper gut microbial balance. This direct role of Duox in gut microbiota control has been demonstrated in studies involving mosquitoes and various other insects [54, 55]. More recently, research has shown that Duox also plays an indirect role in regulating gut symbionts in the bean bug, *Riptortus pedestris* [56]. In this species, Duox expression is specific to the trachea and is essential for maintaining tracheal integrity, which is crucial for supporting mutualistic symbionts.

Duox-derived ROS has been proposed to have a direct bacteria-killing effect in gut immunity and microbiota maintenance [6, 18, 48]. In vitro biochemical evidence supports the role of Duox-derived HOCl but not H_2_O_2_ as the major microbicidal molecule in insect gut immunity [18]. However, Duox has also been suggested to be indirectly involved in gut immunity, possibly as a signaling molecule. For example, Duox-dependent ROS is involved in epithelial cell renewal during gut infection [21, 27, 57] and indispensable for gut muscle contraction and food defecation in *D. melanogaster* during gut infection [16, 17]. These data together suggested a dedicated and controversial role of Duox in insect gut immunity.

The previous conclusion was drawn from a continuous bacterial oral infection, which might be a mixed outcome of both disease resistance and tolerance [18]. Our experiments were done using a short-time oral infection and only focused on the bacteria elimination in the early stage of infection. We found that ROS depletion inhibits bacteria elimination may due to Duox’s role in regulating gut peristalsis, similar to previous findings [16, 17, 22]. More importantly, we found that *Duox* RNAi only slowed down the clearance of bacteria but did not completely inhibit the process. Unlike our observation, it has been reported a prolonged *Ecc15-GFP* persistence in the *Duox* RNAi fly gut at 60 h post-infection, which could be explained by continuous bacteria feeding and long-lasting inhibition of gut peristalsis, as we discussed earlier. Other factors, such as differences in bacterial strains or host species, could also explain the discrepancy. Thus, we propose that early induction of ROS played a major role in supporting gut peristalsis to accelerate food-borne bacteria elimination. However, we cannot exclude the possibility that ROS accumulated in the gut plays a direct bactericidal function, especially under continuous infection. If this is the case, a key question arises: could symbionts, rather than pathobionts, be selectively protected from ROS molecules? Indeed, insect hosts have mechanisms to shield symbionts from damage caused by ROS and reactive nitrogen species. For example, a study in beewolves demonstrated that host-derived hydrocarbons could protect symbionts during transmission from a nitric oxide (NO) burst [58]. Moreover, microorganisms themselves possess redox systems to counteract the effects of ROS [59]. For instance, in bees, the intracellular parasite *Nosema ceranae* utilizes antioxidant systems to adapt to and reproduce within the midgut epithelium [60].

In conclusion, our study highlighted the different roles of pathobionts and symbionts in gut microbiota homeostasis. Importantly, we have characterized Malpighian tubules as a key organ regulating gut immunity through recognizing bacteria-derived tyramine, providing an example of inter-organ communication in gut immunity. Recently, two papers have shown that gut tumors and Malpighian tubules have complex interactions promoting tumor-induced renal dysfunction [61, 62]. Because Malpighian tubules plays a key role in detoxification and excretion, we expect more of the role of the renal system in host physiology will be revealed in the future.

## Supplementary Material

Supplementary_Figures_wrae221

Supplementary_Table_1_wrae221

Supplementary_Table_2_wrae221

Supplementary_Figure_and_Table_legends_wrae221

## Data Availability

All data is available through Figshare (10.6084/m9.figshare.25339495).
