# Supplementary figures and images for: Pathobiont and symbiont contribute to microbiota homeostasis through Malpighian tubules–gut countercurrent flow in Bactrocera dorsalis
Source: ISME J. 2024 Nov 12;18(1):wrae221. doi: 10.1093/ismejo/wrae221 (PMC11697180; doi:10.1093/ismejo/wrae221)

Supplementary Figure 1

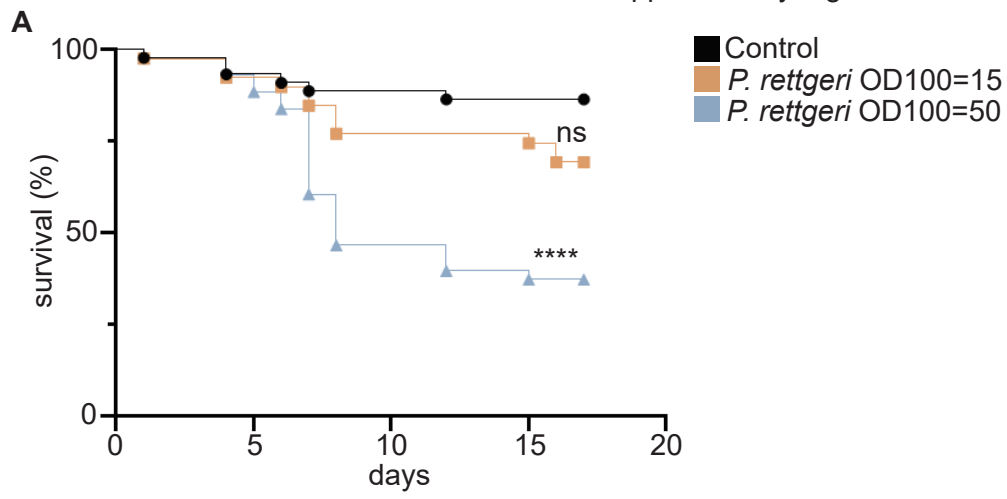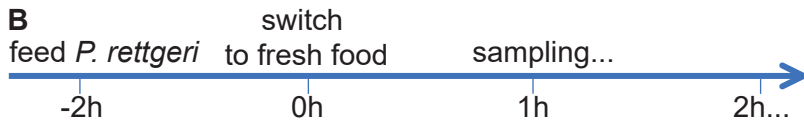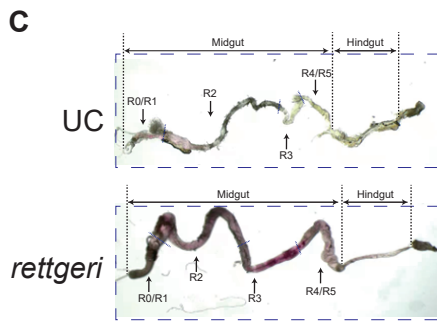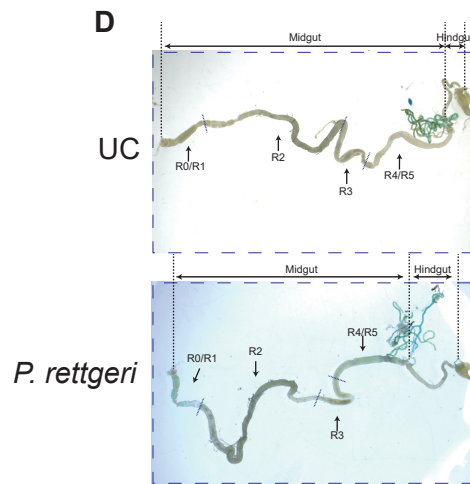

Supplementary Figure 2

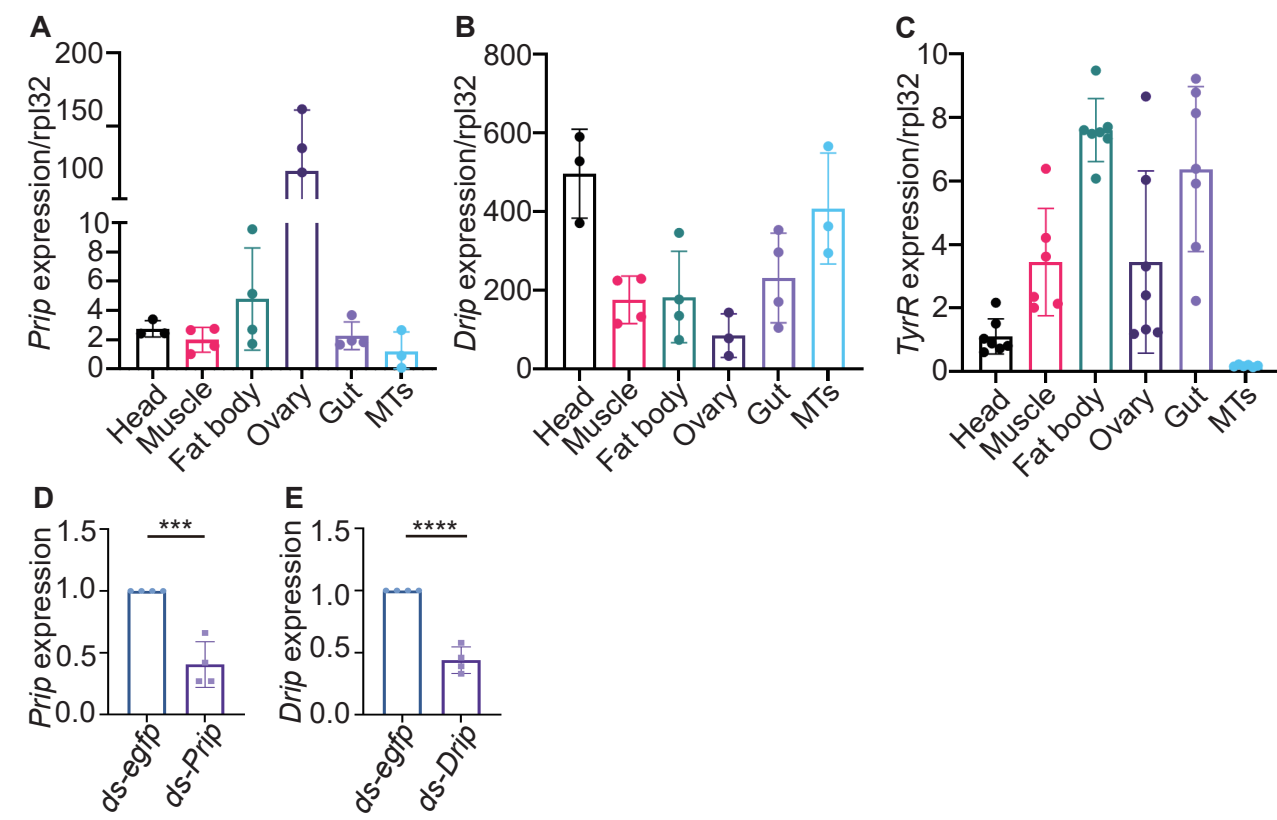

Supplementary Figure 3

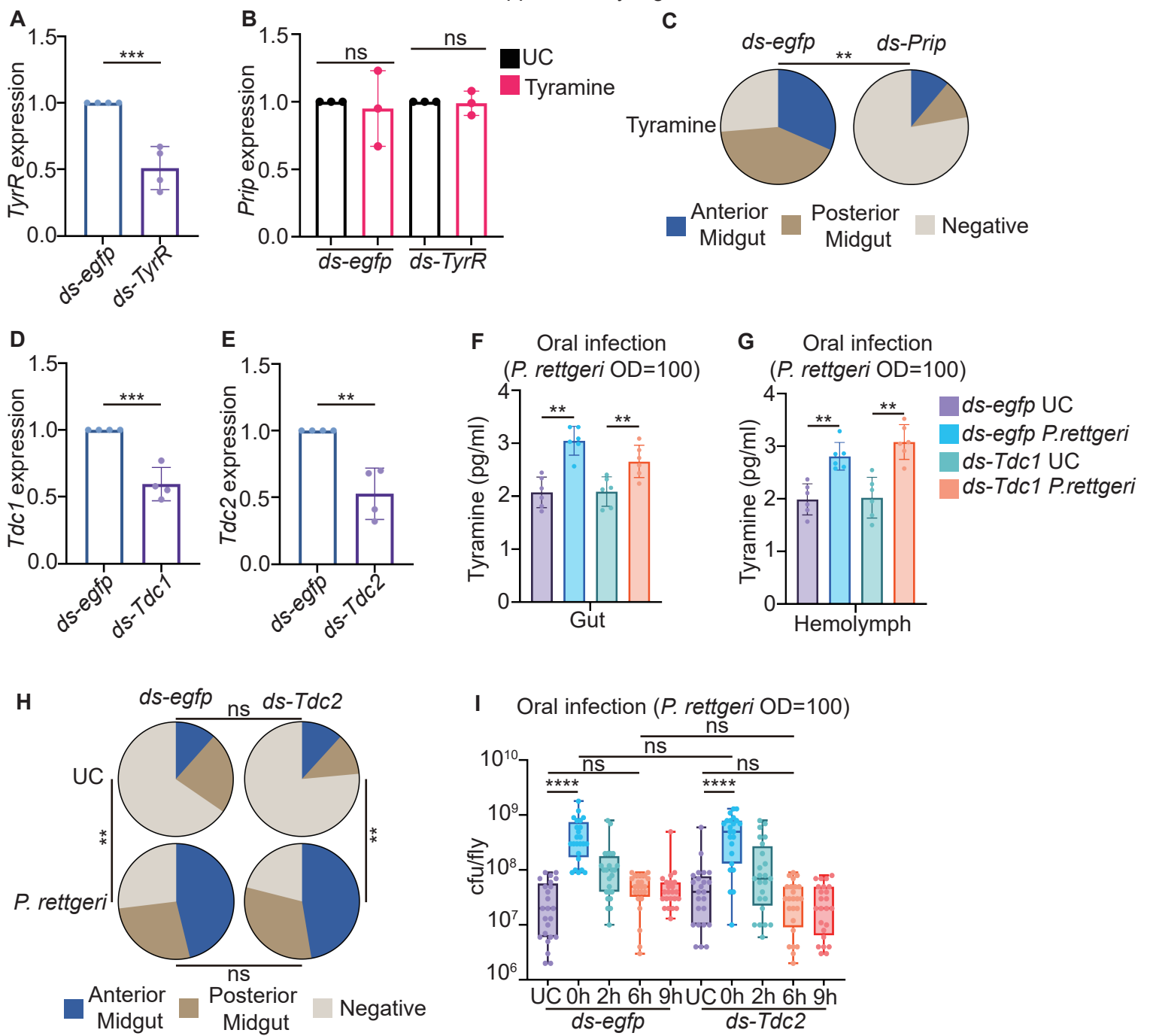

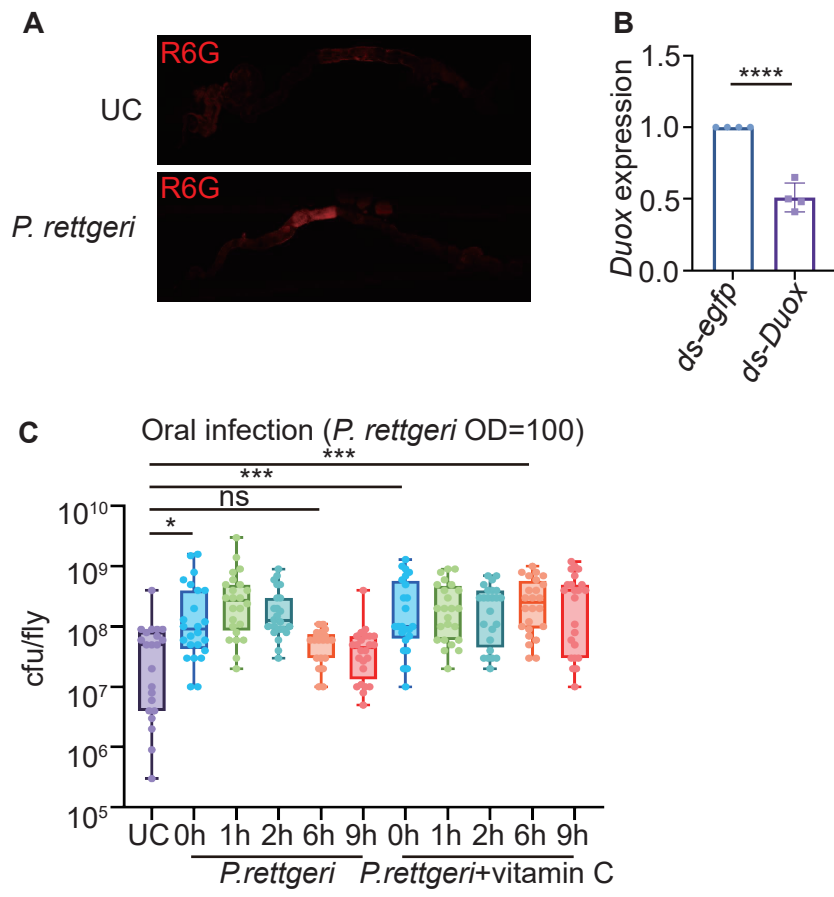

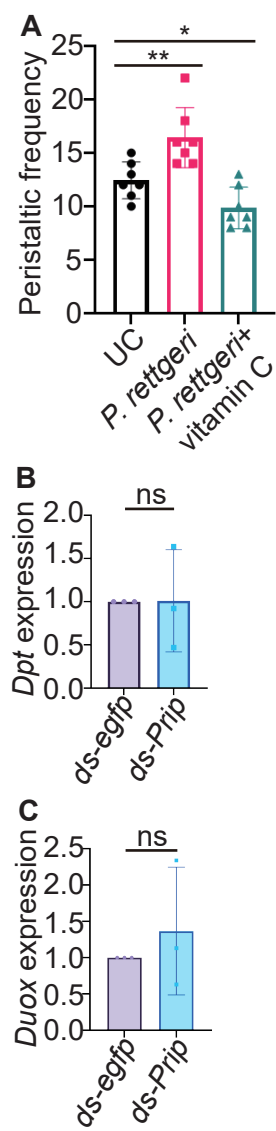

Supplementary Figure 6

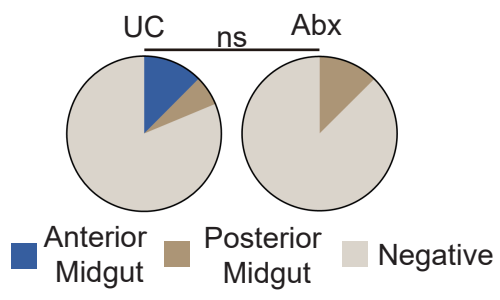

Supplement: Supplementary_Figures_wrae221 [file supplementary_figures_wrae221.pdf]
