# Supplementary material for: Pathobiont and symbiont contribute to microbiota homeostasis through Malpighian tubules–gut countercurrent flow in Bactrocera dorsalis
Source: ISME J. 2024 Nov 12;18(1):wrae221. doi: 10.1093/ismejo/wrae221 (PMC11697180; doi:10.1093/ismejo/wrae221)
Supplement: Supplementary_Table_1_wrae221 [file supplementary_table_1_wrae221.docx]

**Supplementary Table 1**

| **Primers** | **Sequence (5'-3')** |
| --- | --- |
| *Rpl32* QF | CCCGTCATATGCTGCCAACT |
| *Rpl32* QR | GCGCGCTCAACAATTTCCTT |
| *Prip* QF | ATGCACACTGGGTGTACTGG |
| *Prip* QR | ACTCTTCGCTTATCCGCTCA |
| *Prip* T7F | TAATACGACTCACTATAGGGTGTCTATTAAAACGTGTCTCACCT |
| *Prip* T7R | TAATACGACTCACTATAGGGACAATTGCAAGAGCGGAGGA |
| *Drip* QF | TTTGGTCCCGCTGTCATTCA |
| *Drip* QR | GTCCGTCGTCATTTGCAAGC |
| *Drip* T7F | TAATACGACTCACTATAGGGTCGTCGGTGGCAACATCATT |
| *Drip* T7R | TAATACGACTCACTATAGGGGACACACCCAAATTGGTGCC |
| *TyrR* QF | GACGACGTGGCTCCTAAAAC |
| *TyrR* QR | TTCGGAAAGGCCTGAATGCG |
| *TyrR* T7F | TAATACGACTCACTATAGGGTTAGCGTGCGACATCACCAT |
| *TyrR* T7R | TAATACGACTCACTATAGGGTAGGAGCCACGTCGTCTTAC |
| *Tdc1* QF | CCTATGCCGGCAACTCTTTC |
| *Tdc1* QR | GTGCTGATGTCAAGCGGATT |
| *Tdc1* T7F | TAATACGACTCACTATAGGGTCCGCTTGACATCAGCACTT |
| *Tdc1* T7R | TAATACGACTCACTATAGGGCGGATCGCTGACCATTCGTA |
| *Tdc2* QF | ATGGCAAGGATATGGTGGATTAT |
| *Tdc2* QR | TCGGATGGTTCCAATGCACA |
| *Tdc2* T7F | TAATACGACTCACTATAGGGGCATATGCCAGCAAGGAAGC |
| *Tdc2* T7R | TAATACGACTCACTATAGGGTCCTTAACCCACATTGCGGA |
| *Dipterin* QF | GCATAGATTTGAGCCTTGACACAC |
| *Dipterin* QR | GCCATATCGTCCGCCCAAAT |
| *Duox* QF | AGGCATGCGTACTCAACCTA |
| *Duox* QR | TGACAGGCCTTCATCGTCTT |
| *Duox* T7F | TAATACGACTCACTATAGGGACCTGGCGAGTTCTTCACAG |
| *Duox* T7R | TAATACGACTCACTATAGGGCGCGCCAACATAATCTCACG |
| *egfp* T7F | GGATCCTAATACGACTCACTATAGGACGTAAACGGCCACAAGTTC |
| *egfp* T7R | GGATCCTAATACGACTCACTATAGGAAGTCGTGCTGCTTCATGTG |
