# Supplementary material for: Pathobiont and symbiont contribute to microbiota homeostasis through Malpighian tubules–gut countercurrent flow in Bactrocera dorsalis
Source: ISME J. 2024 Nov 12;18(1):wrae221. doi: 10.1093/ismejo/wrae221 (PMC11697180; doi:10.1093/ismejo/wrae221)
Supplement: Supplementary_Table_2_wrae221 [file supplementary_table_2_wrae221.docx]

| **Figures** | **Treatments** | **Statistical methods for data analysis** | ***P* value** | **Adjusted *P* value** | ***P* value summary** |
| --- | --- | --- | --- | --- | --- |
| Figure 1C | UC vs. *P. rettgeri* | chi-square test | 0.028 |  | * |
| Figure 2B | *ds-egfp* UC vs. *ds-egfp* *P. rettgeri* | chi-square test | 0.0392 |  | * |
|  | *ds-egfp* UC vs. *ds-prip* UC | chi-square test | 0.6939 |  | ns |
|  | *ds-prip* UC vs. *ds-prip* *P. rettgeri* | chi-square test | 0.8392 |  | ns |
|  | *ds-egfp* *P. rettgeri* vs. *ds-prip* *P. rettgeri* | chi-square test | 0.0075 |  | ** |
| Figure 2D | *ds-egfp* UC vs. *ds-egfp* *P. rettgeri* | chi-square test | 0.003 |  | ** |
|  | *ds-egfp* UC vs. *ds-drip* UC | chi-square test | 0.8601 |  | ns |
|  | *ds-drip* UC vs. *ds-drip* *P. rettgeri* | chi-square test | 0.0115 |  | * |
|  | *ds-egfp* *P. rettgeri* vs. *ds-drip* *P. rettgeri* | chi-square test | 0.5999 |  | ns |
| Figure 2E | *ds-egfp* UC vs. *ds-egfp* 0h | Kruskal-Wallis tests with Dunn tests | <0.0001 | <0.0001 | **** |
|  | *ds-egfp* UC vs. *ds-egfp* 6h | Kruskal-Wallis test with post-hoc Dunn ’s test | 0.061 | >0.9999 | ns |
|  | *ds-egfp* 0h vs. *ds-prip* 0h | Kruskal-Wallis test with post-hoc Dunn ’s test | 0.4038 | >0.9999 | ns |
|  | *ds-egfp* 6h vs. *ds-prip* 6h | Kruskal-Wallis test with post-hoc Dunn ’s test | 0.0013 | 0.003 | ** |
|  | *ds-prip* UC vs. *ds-prip* 0h | Kruskal-Wallis test with post-hoc Dunn ’s test | <0.0001 | <0.0001 | **** |
|  | *ds-prip* UC vs. *ds-prip* 9h | Kruskal-Wallis test with post-hoc Dunn ’s test | 0.0002 | 0.0075 | ** |
| Figure 2F | *ds-egfp* UC vs. *ds-egfp* 0h | Kruskal-Wallis test with post-hoc Dunn ’s test | <0.0001 | 0.0004 | **** |
|  | *ds-egfp* UC vs. *ds-egfp* 6h | Kruskal-Wallis test with post-hoc Dunn ’s test | 0.2165 | >0.9999 | ns |
|  | *ds-egfp* 0h vs. *ds-drip* 0h | Kruskal-Wallis test with post-hoc Dunn ’s test | 0.6038 | >0.9999 | ns |
|  | *ds-egfp* 6h vs. *ds-drip* 6h | Kruskal-Wallis test with post-hoc Dunn ’s test | 0.54 | >0.9999 | ns |
|  | *ds-drip* UC vs. *ds-drip* 0h | Kruskal-Wallis test with post-hoc Dunn ’s test | <0.0001 | <0.0001 | *** |
|  | *ds-drip* UC vs. *ds-drip* 6h | Kruskal-Wallis test with post-hoc Dunn ’s test | 0.1421 | >0.9999 | ns |
| Figure 3B | *ds-egfp* UC vs. *ds-egfp* *P. rettgeri* | chi-square test | 0.015 |  | * |
|  | *ds-egfp* UC vs. *ds-TyrR* UC | chi-square test | 0.7047 |  | ns |
|  | *ds-TyrR* UC vs. *ds-TyrR* *P. rettgeri* | chi-square test | 0.6168 |  | ns |
|  | *ds-egfp* *P. rettgeri* vs. *ds-TyrR* *P. rettgeri* | chi-square test | 0.0134 |  | * |
| Figure 3C | *ds-egfp* UC vs. *ds-egfp* 0h | Kruskal-Wallis test with post-hoc Dunn ’s test | <0.0001 | <0.0001 | **** |
|  | *ds-egfp* UC vs. *ds-egfp* 6h | Kruskal-Wallis test with post-hoc Dunn ’s test | 0.3635 | >0.9999 | ns |
|  | *ds-egfp* 0h vs. *ds-TyrR* 0h | Kruskal-Wallis test with post-hoc Dunn ’s test | 0.3274 | >0.9999 | ns |
|  | *ds-egfp* 6h vs. *ds-TyrR* 6h | Kruskal-Wallis test with post-hoc Dunn ’s test | 0.0016 | 0.0094 | ** |
|  | *ds-TyrR* UC vs. *ds-TyrR* 0h | Kruskal-Wallis test with post-hoc Dunn ’s test | <0.0001 | <0.0001 | **** |
|  | *ds-TyrR* UC vs. *ds-TyrR* 9h | Kruskal-Wallis test with post-hoc Dunn ’s test | 0.0027 | 0.0164 | ** |
| Figure 3E | Sucrose vs. Tyramine | chi-square test | 0.0352 |  | * |
| Figure 3F | UC vs. 0h(*P. rettgeri*) | Kruskal-Wallis test with post-hoc Dunn ’s test | <0.0001 | <0.0001 | **** |
|  | UC vs. 1h(*P. rettgeri*) | Kruskal-Wallis test with post-hoc Dunn ’s test | <0.0001 | <0.0001 | **** |
|  | UC vs. 1h(*P. rettgeri*+ tyramine) | Kruskal-Wallis test with post-hoc Dunn ’s test | 0.09067 | >0.9999 | ns |
| Figure 3G | Ingested bacteria vs. Whole fly | Mann Whitney U test | 0.0095 |  | * |
|  | Ingested bacteria vs. Gut+MTs | Mann Whitney U test | 0.0095 |  | * |
|  | Ingested bacteria vs. Hemolymph | Mann Whitney U test | 0.0095 |  | * |
| Figure 3I | *ds-egfp* UC vs. *ds-egfp* *P. rettgeri* | chi-square test | 0.0414 |  | * |
|  | *ds-egfp* UC vs. *ds-Tdc1* UC | chi-square test | 0.5965 |  | ns |
|  | *ds-Tdc1* UC vs. *ds-Tdc1* *P. rettgeri* | chi-square test | 0.0752 |  | ns |
|  | *ds-egfp* *P. rettgeri* vs. *ds-Tdc1* *P. rettgeri* | chi-square test | 0.6917 |  | ns |
| Figure 3J | *ds-egfp* UC vs. *ds-egfp* 0h | Kruskal-Wallis test with post-hoc Dunn ’s test | <0.0001 | <0.0001 | **** |
|  | *ds-egfp* UC vs. *ds-egfp* 6h | Kruskal-Wallis test with post-hoc Dunn ’s test | 0.2783 | >0.9999 | ns |
|  | *ds-egfp* 0h vs. *ds-Tdc1* 0h | Kruskal-Wallis test with post-hoc Dunn ’s test | 0.205 | >0.9999 | ns |
|  | *ds-egfp* 6h vs. *ds-Tdc1* 6h | Kruskal-Wallis test with post-hoc Dunn ’s test | 0.4376 | >0.9999 | ns |
|  | *ds-Tdc1* UC vs. *ds-Tdc1* 0h | Kruskal-Wallis test with post-hoc Dunn ’s test | <0.0001 | <0.0001 | **** |
|  | *ds-Tdc1* UC vs. *ds-Tdc1* 6h | Kruskal-Wallis test with post-hoc Dunn ’s test | 0.194 | >0.9999 | ns |
| Figure 4B | UC vs. *P. rettgeri* | Mann Whitney U test | 0.0653 |  | ns |
| Figure 4C | UC vs. *P. rettgeri* 2h | Mann Whitney U test | 0.3214 |  | ns |
| Figure 4F | *ds-egfp* UC vs. *ds-egfp* 0h | Kruskal-Wallis test with post-hoc Dunn ’s test | <0.0001 | <0.0001 | **** |
|  | *ds-egfp* UC vs. *ds-egfp* 6h | Kruskal-Wallis test with post-hoc Dunn ’s test | 0.371 | >0.9999 | ns |
|  | *ds-egfp* 0h vs. *ds-Duox* 0h | Kruskal-Wallis test with post-hoc Dunn ’s test | 0.4964 | >0.9999 | ns |
|  | *ds-egfp* 6h vs. *ds-Duox* 6h | Kruskal-Wallis test with post-hoc Dunn ’s test | <0.0001 | 0.0026 | ** |
|  | *ds-Duox* UC vs. *ds-Duox* 0h | Kruskal-Wallis test with post-hoc Dunn ’s test | <0.0001 | <0.0001 | **** |
|  | *ds-Duox* UC vs. *ds-Duox* 6h | Kruskal-Wallis test with post-hoc Dunn ’s test | 0.0251 | 0.0005 | *** |
| Figure 5A | UC vs. *P. rettgeri* | Mann Whitney U test | 0.0058 |  | ** |
| Figure 5B | *ds-egfp* UC vs. *ds-egfp* *P. rettgeri* | Mann Whitney U test | 0.0022 |  | ** |
|  | *ds-Duox* UC vs. *ds-Duox* *P. rettgeri* | Mann Whitney U test | 0.1126 |  | ns |
| Figure 5G | *ds-egfp* UC vs. *ds-egfp* *P. rettgeri* | Mann Whitney U test | 0.0079 |  | ** |
|  | *ds-prip* UC vs. *ds-prip* *P. rettgeri* | Mann Whitney U test | 0.5159 |  | ns |
| Figure 6B | UC vs. *C. koseri* | chi-square test | 0.0205 |  | * |
|  | UC vs. *E. hormaechei* | chi-square test | 0.0302 |  | * |
|  | *C. koseri* vs. *E. hormaechei* | chi-square test | 0.8487 |  | ns |
| Supplementary Figure 1A | Control vs. OD100=15 | Log-rank (Mantel-Cox) test | 0.742 |  | ns |
|  | Control vs. OD100=50 | Log-rank (Mantel-Cox) test | <0.0001 |  | **** |
| Supplementary Figure 2D | *ds-egfp* vs. *ds-Prip* | Mann Whitney U test | 0.0007 |  | *** |
| Supplementary Figure 2E | *ds-egfp* vs. *ds-Drip* | Mann Whitney U test | <0.0001 |  | **** |
| Supplementary Figure 3A | *ds-egfp* vs. *ds-TyrR* | Mann Whitney U test | 0.0009 |  | *** |
| Supplementary Figure 3B | *ds-egfp* UC vs. *ds-egfp* tyramine | Mann Whitney U test | 0.7725 |  | ns |
|  | *ds-TyrR* UC vs. *ds-TyrR* tyramine | Mann Whitney U test | 0.8568 |  | ns |
| Supplementary Figure 3C | *ds-egfp* tyramine vs. *ds-prip* tyramine | chi-square test | 0.0073 |  | ** |
| Supplementary Figure 3D | *ds-egfp* vs. *ds-Tdc1* | Mann Whitney U test | 0.0006 |  | *** |
| Supplementary Figure 3E | *ds-egfp* vs. *ds-Tdc2* | Mann Whitney U test | 0.0026 |  | ** |
| Supplementary Figure 3F | *ds-egfp* UC vs. *ds-egfp* *P. rettgeri* | Mann Whitney U test | 0.0022 |  | ** |
|  | *ds-Tdc1* UC vs. *ds-Tdc1* *P. rettgeri* | Mann Whitney U test | 0.0087 |  | ** |
| Supplementary Figure 3G | *ds-egfp* UC vs. *ds-egfp* *P. rettgeri* | Mann Whitney U test | 0.0022 |  | ** |
|  | *ds-Tdc1* UC vs. *ds-Tdc1* *P. rettgeri* | Mann Whitney U test | 0.0022 |  | ** |
| Supplementary Figure 3H | *ds-egfp* UC vs. *ds-egfp* *P. rettgeri* | chi-square test | 0.0081 |  | ** |
|  | *ds-egfp* UC vs. *ds-Tdc2* UC | chi-square test | 0.6413 |  | ns |
|  | *ds-Tdc2* UC vs. *ds-Tdc2* *P. rettgeri* | chi-square test | 0.0038 |  | ** |
|  | *ds-egfp* *P. rettgeri* vs. *ds-Tdc2* *P. rettgeri* | chi-square test | 0.8867 |  | ns |
| Supplementary Figure 3I | *ds-egfp* UC vs. *ds-egfp* 0h | Kruskal-Wallis test with post-hoc Dunn ’s test | <0.0001 | <0.0001 | **** |
|  | *ds-egfp* UC vs. *ds-egfp* 6h | Kruskal-Wallis test with post-hoc Dunn ’s test | 0.0648 | 0.5183 | ns |
|  | *ds-egfp* 0h vs. *ds-Tdc2* 0h | Kruskal-Wallis test with post-hoc Dunn ’s test | 0.6958 | >0.9999 | ns |
|  | *ds-egfp* 6h vs. *ds-Tdc2* 6h | Kruskal-Wallis test with post-hoc Dunn ’s test | 0.1138 | 0.9108 | ns |
|  | *ds-Tdc2* UC vs. *ds-Tdc2* 0h | Kruskal-Wallis test with post-hoc Dunn ’s test | <0.0001 | <0.0001 | **** |
|  | *ds-Tdc2* UC vs. *ds-Tdc2* 6h | Kruskal-Wallis test with post-hoc Dunn ’s test | 0.2914 | >0.9999 | ns |
| Supplementary Figure 4B | *ds-egfp* vs. *ds-Duox* | Mann Whitney U test | <0.0001 |  | **** |
| Supplementary Figure 4C | UC vs. 0h (*P. rettgeri*) | Kruskal-Wallis test with post-hoc Dunn ’s test | 0.0025 | 0.01 | ** |
|  | UC vs. 6h (*P. rettgeri*) | Kruskal-Wallis test with post-hoc Dunn ’s test | 0.4412 | >0.9999 | ns |
|  | UC vs. 0h (*P. rettgeri*+ VC) | Kruskal-Wallis test with post-hoc Dunn ’s test | 0.0002 | 0.0007 | *** |
|  | UC vs. 9h (*P. rettgeri*+ VC) | Kruskal-Wallis test with post-hoc Dunn ’s test | <0.0001 | 0.0002 | *** |
| Supplementary Figure 5A | UC vs. *P. rettgeri* | Mann Whitney U test | 0.0058 |  | ** |
|  | UC vs. *P. rettgeri*+ *Vitamin C* | Mann Whitney U test | 0.0367 |  | * |
| Supplementary Figure 5B | *ds-egfp* vs. *ds-Prip* | Mann Whitney U test | 0.978 |  | ns |
| Supplementary Figure 5C | *ds-egfp* vs. *ds-Prip* | Mann Whitney U test | 0.5101 |  | ns |
| Supplementary Figure 6A | UC vs. Abx | chi-square test | 0.3057 |  | ns |
