# Supplementary material for: Pathobiont and symbiont contribute to microbiota homeostasis through Malpighian tubules–gut countercurrent flow in Bactrocera dorsalis
Source: ISME J. 2024 Nov 12;18(1):wrae221. doi: 10.1093/ismejo/wrae221 (PMC11697180; doi:10.1093/ismejo/wrae221)
Supplement: Supplementary_Figure_and_Table_legends_wrae221 [file supplementary_figure_and_table_legends_wrae221.docx]

**Supplementary Figure 1.**

**(A) Survival of *B. doralis* infected by different *P. rettgeri* concentration.** Flies were fed on food completely covered by a filter paper disk containing *P. rettgeri* (OD 600 = 15 and 50). The data represent the average of 3 biological replicates. *****P* value < 0.0001; ns non-significant. Log-rank (Mantel-Cox) test, see detailed *P* values in Supplementary Table 2.

**(B) Schematic diagram of experimental setup.** The female orange fly was starved for 2 hours in advance and began to feed *P. rettgeri*. After 2 hours feeding, the bacterial solution was replaced with fly food. This time point was set to 0h for sampling in subsequent experiments.

**(C) *in vivo* countercurrent flow experiment using Amaranth red injection.**

**(D) Dye-absorption in the tubules before and after infection.**

**Supplementary Figure 2.**

**(A-B) Expression profiles of *Prip* and *Drip* in different tissues of *B. dorsalis.***

**(C) Expression profiles of** ***TyrR* and *Drip* in different tissues of *B. dorsalis.***

**(D-E) RNA interference efficiency of *Prip* and *Drip.*** For RNAi efficiency RT-qPCR, each data point represents result from a pooled sample of 8 flies. For expression profiles, each data point represents result from a pooled sample of 20 flies. ****P* value < 0.001; *****P* value < 0.0001. Mann-Whitney U test, see detailed *P* values in Supplementary Table 2.

**Supplementary Figure 3.**

**(A) RNA interference efficiency of *TyrR.***

**(B) *TyrR* RNAi and Tyramine feeding did not affect *Prip* expression.**

**(C) Tyramine feeding did not affect Malpighian tubules-gut countercurrent flow in *Prip* RNAi flies.** *egfp* RNAi flies were used as control. (n=18-19).

**(D-E) RNA interference efficiency of *Prip* and *Drip.***

**(F and G) Contents of tyramine in gut and hemolymph after oral infection of *Tdc1* RNAi.** The contents of tyramine in gut and hemolymph increased significantly after oral infection of *Tdc1* RNAi. *egfp* RNAi flies as control. UC: unchallenged.

**(H-I) *Tdc2* RNAi did not affect Malpighian tubules-gut countercurrent flow and bacteria elimination.** Silencing *Tdc2* did not affect the formation of Malpighian tubules-gut countercurrent flow (H). *Tdc2* RNAi did not affect the clearance of *P. rettgeri* in the gut of *B.dorsalis* (I). *egfp* RNAi flies were used as control. (n=17-26 for (H), n=24 for (I)). Each data point represents bacteria burden from a single fly. For For RNAi efficiency RT-qPCR,RT-qPCR, each data point represents result from a pooled sample of 8 flies. UC: unchallenged. ***P* value <0.01; ****P* value < 0.001; *****P* value < 0.0001; ns non-significant. A, B, D, E, F, and G were analyzed using Mann-Whitney U test, C and H were analyzed using chi-square test, I was analyzed using Kruskal-Wallis test with post-hoc Dunn ’s test, see detailed *P* values in Supplementary Table 2.

**Supplementary Figure 4.**

**(A) HOCl staining in the whole gut after *P. rettgeri* infection.**

**(B) RNA interference efficiency of *Duox.*** Each data point represents result from a pooled sample of 8 flies.

**(C) Co-fed antioxidant** vitamin C **with bacteria delayed *B. dorsalis* bacteria elimination.** *P. rettgeri*-fed were used as control. (n=21-24). UC: unchallenged, **P* value < 0.05; ****P* value < 0.001; *****P* value < 0.0001; ns non-significant. B was analyzed using Mann-Whitney U test, C was analyzed using Kruskal-Wallis test with post-hoc Dunn’s test, see detailed *P* values in Supplementary Table 2.

**Supplementary Figure 5.**

**(A) Vitamin C feeding reduces the frequency of gut peristalsis.**

**(B and C) Effects of *Prip* silencing on ROS and AMP production.** *Prip* RNAi had no effect on the expression of *Duox* and *dpt*. The expression of *Duox* and *dpt* genes were normalized into *egfp* RNAi group, which was set as 1. *egfp* RNAi was used as a control. Each data point represents result from a pooled sample of 20 flies (n=3). UC: unchallenged. **P* value < 0.1; ***P* value < 0.01; ns non-significant. A, B, and C were analyzed using Mann-Whitney U test, see detailed *P* values in Supplementary Table 2.

**Supplementary Figure 6.**

**Antibiotic feeding did not affect Malpighian tubules-gut countercurrent flow.** (n=16). UC: unchallenged. ns non-significant. Chi-square test, see detailed *P* values in Supplementary Table 2.

**Supplementary Table 1.**

**The primers used in the article.** Primers for RT-qPCR and dsRNA synthesis are listed in the table.

**Supplementary Table 2.**

**Statistical analysis of the data presented in the paper.** Statistical methods for data analysis, un-adjusted *P* value, adjusted *P* value and *P* value summary for all data are listed in the table.
